# Multidimensional Frailty and Resilience in Relation to Quality of Life in Senior Patients with Cardiovascular Disease: An Exploratory Cross-Sectional Pilot Study

**DOI:** 10.3390/jcm15156083

**Published:** 2026-08-05

**Authors:** Sabinne-Marie Albișteanu, Bianca Nistoreanu-Neculau, Alice Nistoreanu-Neculau, Ramona Ștefăniu, Gabriela Grigoraș, Iulia-Daniela Lungu, Diana-Gabriela Constantinescu, Ana-Maria Turcu, Adina-Carmen Ilie, Anca Iuliana Pîslaru

**Affiliations:** 1 Grigore T. Popa University of Medicine and Pharmacy, 700115 Iași, Romania; 2 Faculty of Psychology and Educational Sciences, “Alexandru Ioan Cuza” University of Iași, 700506 Iași, Romania; 3 CETI Day Center for the Rehabilitation of Children with Special Needs, 7 Ștefan O. Iosif Alley, 700273 Iași, Romania

**Keywords:** multidimensional frailty, psychological resilience, quality of life, comprehensive geriatric assessment, cardiovascular disease, elderly

## Abstract

**Background/Objectives:** Cardiovascular diseases are the leading cause of morbidity and mortality in older adults and are frequently associated with multidimensional frailty and reduced quality of life. The present study aimed to investigate the associations among multiple domains of the Comprehensive Geriatric Assessment (CGA), including frailty, cognitive status, emotional status, nutritional status, functional status, fall risk, psychological resilience, and perceived quality of life, in older adults with cardiovascular disease. **Methods:** A cross-sectional observational study was conducted on 100 patients aged ≥60 years admitted to the Geriatrics Department of the “Dr. C.I. Parhon” Clinical Hospital, Iași. Cognitive, emotional, nutritional, and functional status, fall risk, frailty (Fried and Tilburg scales), resilience- Connor–Davidson Resilience Scale-25 (CD-RISC-25), and quality of life- World Health Organization Quality of Life–BREF (WHOQOL-BREF) were assessed. Statistical analysis included chi-square or Fisher’s exact tests and exploratory discrimination analysis using ROC curves. **Results:** Overall, 61% of participants reported a very good quality of life, whereas the remaining 39% reported a moderately reduced quality of life. Better perceived quality of life was significantly associated with preserved cognitive status, fewer depressive symptoms, better nutritional status, lower frailty (as assessed by both the Fried Frailty Phenotype and the Tilburg Frailty Indicator), preserved functional independence, lower fall risk, and greater psychological resilience (all *p* < 0.05). In contrast, neither demographic characteristics nor cardiovascular diagnoses were significantly associated with quality of life. Exploratory receiver operating characteristic (ROC) analysis demonstrated modest discriminatory performance for cognitive status— Mini-Mental State Examination (MMSE) (AUC = 0.660; *p* = 0.007), nutritional status— Mini Nutritional Assessment (MNA) (AUC = 0.624; *p* = 0.038), and the Tilburg Frailty Indicator (AUC = 0.635; *p* = 0.024), whereas psychological resilience exhibited an inverse relationship with the predefined outcome (AUC = 0.293; *p* = 0.001). **Conclusions:** In hospitalized older adults with cardiovascular disease, perceived quality of life was primarily associated with multidimensional geriatric, functional, nutritional, cognitive, and psychosocial factors, rather than with demographic characteristics or cardiovascular diagnoses. Psychological resilience was identified as an important psychosocial correlate of better perceived quality of life, supporting its inclusion in individualized comprehensive geriatric interventions. These findings highlight the importance of comprehensive geriatric assessment in identifying factors associated with quality of life. Given the cross-sectional design, the observed relationships should be interpreted as associations rather than causal effects and require confirmation in prospective longitudinal studies.

## 1. Introduction

Cardiovascular diseases remain the leading cause of morbidity and mortality worldwide, accounting for approximately 17.9 million deaths annually (around 32% of global mortality), with a substantially increased burden in the elderly population [1]. Despite advances in therapeutic approaches and modern preventive strategies, they continue to represent a major cause of functional decline, recurrent hospitalizations, disability, and deterioration of quality of life in older adults [1,2,3].

Frailty, defined across its multiple dimensions (physical, cognitive, psycho-emotional, and social), represents an important determinant of prognosis in older patients with cardiovascular disease and a risk factor for cardiovascular pathology itself. Its prevalence is increased in hypertension, chronic coronary syndromes, atrial fibrillation, and heart failure, where it serves as a marker of clinical vulnerability with an impact on therapeutic decision-making and long-term outcomes [4,5,6,7].

In older patients, hypertension, chronic coronary syndrome, atrial fibrillation, and heart failure share common pathophysiological mechanisms with frailty, including chronic inflammation, oxidative stress, endothelial dysfunction, neurohormonal activation, and sarcopenia. In hypertension, the presence of frailty modifies cardiovascular risk and therapeutic response, requiring individualized management. In chronic coronary syndrome, frailty is associated with cognitive impairment, depression, polypharmacy, and functional decline. In atrial fibrillation, it increases the risk of falls and bleeding, limits pharmacological and interventional therapeutic options due to heightened susceptibility to iatrogenesis, and reduces the feasibility of optimal anticoagulation, both in relation to socioeconomic status and the need for continuous reassessment of the risk-benefit balance. In heart failure, a progressive geriatric syndrome, frailty overlaps with the disease, sharing common clinical manifestations, amplifies functional and psychosocial vulnerability, and is associated with poor prognosis and reduced quality of life [4,5,6,7].

It is important to emphasize that frailty is a dynamic condition, varying along a continuum from robustness to pre-frailty and frailty, thereby providing a “window of intervention” for prevention and potential reversibility. The underlying pathophysiological processes involve impairment of the neuromuscular, endocrine, and immune systems, together with low-grade chronic inflammation (inflammaging), sarcopenia, osteopenia, and nutritional imbalances. In contrast, disability refers to limitations or dependency in activities of daily living and is frequently associated with frailty and comorbidities [8,9].

The result of these definitional overlaps has been the refinement of the concept of frailty, which is now recognized as a multidimensional clinical syndrome of increasing specificity. Frailty assessment can be performed using validated instruments that reflect the complexity of this construct. Thus, the Fried phenotype primarily captures the physical component of frailty, whereas the Tilburg Frailty Indicator provides a multidimensional assessment integrating physical, psychological, and social domains, with its feasibility and ease of administration making it particularly suitable for geriatric populations and patients with cardiovascular disease [10,11,12].

Beyond traditional cardiovascular targets, quality of life has become a central outcome in older patients, reflecting not only symptom burden and physical status, but also cognitive and emotional functioning, as well as the degree of autonomy. In this population, factors associated with frailty have been reported to influence quality of life to a greater extent than conventional cardiological parameters [13,14,15].

There has, however, been a growing scientific interest in recent years in assessing the interdependence between these concepts. In light of ongoing efforts to clarify these relationships, we aim to explore the associations between frailty, resilience and quality of life using standardized instruments within a comprehensive geriatric assessment. This approach integrates all dimensions of the older patient’s profile (cognitive, emotional, nutritional, and functional), which contribute to the multidimensional characterization of frailty and resilience [11,12,13,14,15,16].

Resilience is a construct complementary to frailty, defined as the individual’s ability to adapt and respond to biological and psychological stressors. It encompasses psycho-emotional, cognitive, and physical dimensions, has a multimodal character, and represents a distinct trait rather than the opposite of frailty. In the context of aging and cardiovascular disease, resilience has gained increasing attention as a protective psychosocial factor influencing functional outcomes, symptom perception, and health-related quality of life. Higher levels of resilience are associated with better emotional adaptation, improved treatment adherence, and enhanced quality of life. As a potentially modifiable construct through psychosocial interventions, resilience has been proposed as a promising target for future interventions in older patients with cardiovascular disease [16,17,18].

Based on the available evidence linking frailty and psychosocial factors with quality of life in older adults with cardiovascular disease, we hypothesize that multidimensional frailty and psychological resilience are associated with perceived quality of life, and that resilience may act as a key modulating factor in this relationship.

To our knowledge, this study uniquely integrates multidimensional frailty and psychological resilience as complementary constructs of vulnerability and adaptation, and examines their simultaneous relationship with quality of life in older hospitalized patients with cardiovascular disease. Rather than analyzing these factors in isolation, we conceptualize frailty and resilience as interacting dimensions within a unified geriatric framework. In addition, the combined use of two validated frailty instruments (Fried Frailty phenotype and Tilburg Frailty Indicator) together with a standardized resilience scale provides a more comprehensive assessment of the physical, psychological, and social domains of aging in cardiovascular patients. This integrated approach provides a more nuanced understanding of factors associated with quality of life in a clinically complex population of hospitalized older adults.

The present study aimed to evaluate the relationship among multidimensional frailty, psychological resilience, and quality of life in older patients with cardiovascular disease within the framework of a CGA. We also sought to identify the geriatric and psychosocial variables most strongly associated with quality of life in this patient population. Given the exploratory nature of the study, the findings should be interpreted as preliminary.

## 2. Materials and Methods

### 2.1. Study Design

The present study employed a pilot, observational, exploratory cross-sectional design, aiming to simultaneously assess frailty, resilience, and quality of life in older patients with cardiovascular disease within the context of a comprehensive geriatric assessment.

### 2.2. Study Setting

The study was conducted in the Geriatrics Department of the “Dr. C.I. Parhon” Clinical Hospital in Iași, Romania, between February and April 2025. During this period, eligible patients were consecutively recruited upon hospitalization and included in the final analysis.

All participants underwent a CGA, which included the collection of clinical and demographic data, as well as the administration of a geriatric test battery (MMSE, GDS, MNA, ADL, IADL, Downton Scale) and standardized instruments for the evaluation of frailty, resilience, and quality of life. All questionnaires were administered through standardized face-to-face interviews by trained physicians, ensuring consistent administration across all participants.

### 2.3. Participants

Inclusion criteria:

▪ age ≥ 60 years;
▪ cardiovascular diseases/conditions (hypertension, chronic coronary syndrome, atrial fibrillation, heart failure), established according to current clinical guidelines;
▪ clinically stable status at the time of assessment (in the absence of acute decompensation);
▪ ability to understand and complete the assessment instruments;
▪ signed informed consent for study participation.

Exclusion criteria:

▪ age < 60 years;
▪ presence of hypertensive crisis, acute ischemia, atrial fibrillation with rapid ventricular response, acute heart failure episode, or hemodynamic instability at the time of assessment;
▪ severe cognitive impairment preventing questionnaire completion (e.g., advanced dementia);
▪ refusal to participate or withdrawal of informed consent;
▪ severe acute non-cardiac conditions interfering with assessment (e.g., major acute infections, terminal conditions).

Participants were consecutively recruited based on the aforementioned inclusion and exclusion criteria during hospitalization in the Geriatrics Department. All eligible patients provided informed consent prior to study inclusion.

All assessments were performed under clinically stable conditions during a non-urgent hospitalization within the study period.

In patients with mild cognitive or physical limitations, questionnaires were administered in interview format by trained physicians to reduce missing responses and minimize respondent burden. Patients with severe cognitive impairment were excluded. All assessments were performed during hospitalization in a controlled clinical environment, with assistance provided only for clarification of questionnaire items, without influencing patient responses.

Although hypertension is primarily regarded as a cardiovascular risk factor rather than a clinical entity comparable to heart failure or atrial fibrillation, it was included as a cardiovascular comorbidity due to its high prevalence and clinical relevance in the studied population.

Chronic coronary syndrome (CCS) was defined as stable ischemic heart disease, including patients with a history of stable angina and/or previous myocardial revascularization in a clinically stable condition, consistent with contemporary clinical cardiology classifications. This terminology is consistent with standard clinical practice in contemporary cardiology.

### 2.4. Response Rate

Of the 100 eligible patients consecutively admitted during the study period, all were enrolled and completed the baseline clinical assessment under supervision. Questionnaires were administered face to face by trained clinicians in a controlled inpatient setting, which allowed immediate clarification of items and verification of completeness. This procedure resulted in no missing data for the final analyzed dataset. This was facilitated by real-time supervision during face-to-face administration, allowing immediate resolution of comprehension issues and ensuring completeness of responses.

### 2.5. Methods

The methods described below were designed to achieve the study objectives outlined in the introduction.

A comprehensive geriatric assessment was performed in older patients with cardiovascular disease, including a geriatric test battery as well as the assessment of quality of life, resilience, and frailty using internationally validated instruments.

▪ Cognitive status

Cognitive status was assessed using the Mini-Mental State Examination (MMSE), a 30-point questionnaire evaluating orientation, memory, attention, language, and visuospatial abilities, developed by Folstein et al. MMSE scores range from 0 to 30, with scores ≥25 indicating normal cognitive function, 20–24 mild impairment, 10–19 moderate impairment, and <10 severe impairment. A score <25 was considered suggestive of cognitive impairment.

▪ Psycho-emotional status

Depressive symptoms were assessed using the Geriatric Depression Scale (GDS), a validated instrument designed for the older population. The score ranges from 0 to 15 and is interpreted as follows: 0–4 normal mood status, 5–11 mild depression, and 12–15 severe depression. A score >5 was used to identify the presence of depressive symptoms.

▪ Nutritional status

Nutritional status was evaluated using the Mini Nutritional Assessment (MNA), which assesses dietary intake, weight loss, mobility, psychological stress, and body mass index. The total score ranges from 0 to 30, with values ≥24 indicating normal nutritional status, 17–23.5 indicating risk of malnutrition, and <17 indicating malnutrition. A score <23.5 was considered suggestive of nutritional risk.

▪ Functional status

Functional status was assessed using the Activities of Daily Living (ADL) scale and the Instrumental Activities of Daily Living (IADL) scale.

ADL was used to evaluate independence in basic activities (personal hygiene, dressing, feeding, transfers, toileting, and continence), with higher scores indicating a greater level of functional independence.

IADL was used to assess more complex activities (telephone use, shopping, meal preparation, medication management, financial handling, and domestic tasks). Higher scores (>3 on both scales) indicate better functional autonomy in daily life.

▪ Fall risk

Fall risk was assessed using the Downton Scale, a validated instrument that includes risk factors such as previous falls, medication use, sensory deficits, mental status, and mobility.

The total score is obtained by summing individual items, and a score ≥2 indicates an increased risk of falls.

▪ Quality of life

Quality of life was assessed using the World Health Organization Quality of Life-BREF (WHOQOL-BREF), an instrument developed by the World Health Organization (WHO Programme on Mental Health, Geneva). This is a widely used research tool designed to evaluate individuals’ subjective perception of their quality of life and overall health status. The questionnaire comprises 26 items, including two items assessing overall quality of life and general health satisfaction, while the remaining 24 items are organized into four domains: physical health, psychological health, social relationships, and environment.

The physical health domain (D1) assesses facets such as energy and fatigue, pain and discomfort, sleep and rest, mobility, activities of daily living, and dependence on medical treatment or assistance. The psychological domain (D2) covers positive and negative feelings, self-esteem, body image, concentration, and personal beliefs, including spirituality. The social relationships domain (D3) evaluates personal relationships, social support, and sexual activity. The environment domain (D4) includes financial resources, safety, access to health and social care, home environment, opportunities for acquiring information and skills, participation in recreation and leisure activities, transport, and aspects of the physical environment such as pollution, noise, traffic, and climate [19].

Responses are recorded on a 5-point Likert scale, and domain scores are calculated as means, with higher scores indicating better quality of life.

For the purpose of this study, domain scores were calculated according to WHOQOL-BREF guidelines, and a global quality-of-life score was derived as the sum of the four domain scores (physical, psychological, social, and environmental). Although WHOQOL-BREF is primarily designed for domain-specific interpretation, a global score was used in this study to facilitate an overall comparative analysis of quality of life within the cohort (Table S1-Supplementary Materials).

Participants were categorized into two groups based on the sample mean of the global QoL score, due to the absence of universally accepted cut-off values for WHOQOL-BREF global scoring. This dichotomization was performed for exploratory purposes to enable group-based comparisons in this cross-sectional study and was not intended for clinical classification.

▪ Resilience

Resilience was assessed using the Connor–Davidson Resilience Scale (CD-RISC-25). The instrument comprises 25 items rated on a 5-point Likert scale (0–4), yielding a total score ranging from 0 to 100.

Higher scores indicate greater levels of resilience.

▪ Frailty

Frailty was assessed using two complementary instruments.

Fried Phenotype

Physical frailty was assessed according to the Fried phenotype, based on five validated criteria: unintentional weight loss, self-reported exhaustion, low physical activity, slow gait speed, and reduced grip strength. Each component was operationalized using standardized thresholds as originally described by Fried et al. [10].

Participants were classified into three categories:

Robust (0 criteria);

Pre-frail (1–2 criteria);

Frail (≥3 criteria).

Grip strength was assessed using a Jamar Hydraulic Hand Dynamometer (Performance Health, Warrenville, IL, USA), adjusted for sex and body mass index. Gait speed was measured over a standardized walking distance, with cut-off values adjusted for height and sex. Exhaustion and physical activity were assessed using validated questionnaire items, and unintentional weight loss was based on self-reported history.

For statistical analysis, all three categories (robust, pre-frail, frail) were retained and analyzed separately, unless otherwise specified in regression models where categories were grouped to ensure statistical stability.

Tilburg Frailty Indicator (TFI)

The Tilburg Frailty Indicator (TFI) was used as a multidimensional measure of frailty, consisting of 15 items covering physical, psychological, and social domains. The total score ranges from 0 to 15, with higher scores indicating greater frailty severity [11].

In this study, TFI was analyzed both as a continuous variable (mean ± SD) and as a categorical variable based on validated thresholds (non-frail vs frail). In addition, the full score distribution was reported to ensure transparency of frailty severity within the study population.

Frailty distribution

According to the Fried phenotype, participants were distributed across robust, pre-frail, and frail categories, with a predominance of frail and pre-frail individuals. The Tilburg Frailty Indicator showed a broad distribution of scores ranging from X to Y (mean ± SD), reflecting varying degrees of multidimensional frailty within the study population.

Integrated analysis

The results were interpreted within a multidimensional geriatric assessment framework, integrating physical, psychological, and social domains to characterize the interaction between frailty, resilience, and quality of life. All variables were fully collected; no missing data were recorded.

### 2.6. Work Stages

1. Establishment of inclusion and exclusion criteria and development of the study protocol.
2. Submission and approval of the study protocol by the Ethics Committee of the “Dr. C. I. Parhon” Clinical Hospital, Iași, including all study-related documents.
3. Preparation and finalization of assessment instruments and methodological design of the comprehensive geriatric evaluation.
4. Development of the informed consent forms in accordance with the approved ethical framework and institutional requirements.
5. Recruitment of participants and obtaining written informed consent prior to inclusion.
6. Administration of the comprehensive geriatric assessment and the instruments for evaluating quality of life, frailty, and resilience, as well as data collection, ensuring anonymization and confidentiality of information.

### 2.7. Parameters Studied

The following parameters were analyzed to assess associations with frailty, resilience, and quality of life in older patients with cardiovascular disease:

1. Demographic parameters: age, sex, place of residence, marital status.
2. Functional and geriatric status: functional capacity (ADL, IADL), cognitive status (MMSE), affective status (GDS), nutritional status (MNA), and fall risk (Downton Scale).
3. Frailty: assessed as a multidimensional phenomenon (TFI and Fried Phenotype).
4. Resilience: total CD-RISC-25 score.
5. Quality of life: WHOQOL-BREF domain scores (physical, psychological, social, and environmental domains).

### 2.8. Data Sources and Measurement

All assessments were standardized across participants using validated instruments.

### 2.9. Bias Control

Selection bias was minimized through consecutive recruitment and clear criteria.

Information bias was reduced by using the same team for all measurements. All questionnaires were administered face-to-face in a standardized interview format by trained physicians in a quiet inpatient setting, with items read aloud to participants when necessary and responses recorded verbatim. The administration followed a uniform script to ensure consistency across patients.

Because of the cross-sectional design, temporal relationships between variables could not be established.

The absence of missing data is explained by the supervised administration of all questionnaires during hospitalization, where trained investigators ensured completeness at the time of data collection. Patients with cognitive or physical limitations were assisted in completing the instruments when needed, without influencing their responses.

Capacity to provide informed consent was assessed clinically by the attending physician prior to inclusion. Patients with severe cognitive impairment or inability to understand study procedures were excluded. Participants with mild to moderate cognitive impairment who demonstrated sufficient comprehension were included, and informed consent was obtained directly from them. In cases of cognitive difficulty, questionnaire items were administered using structured interviewer assistance to ensure comprehension, without modifying or suggesting responses.

### 2.10. Sample Size

The study included all eligible patients available during the recruitment period (*n* = 100). No formal statistical sample size calculation or power analysis was performed prior to data collection, as all available participants were included. Therefore, the findings should be considered exploratory and hypothesis-generating rather than confirmatory. This approach ensured that the study captured the full population of interest within the selected department.

### 2.11. Quantitative Variables

Variables were analyzed as continuous or categorized per clinical and scientific guidelines.

### 2.12. Statistical Analysis

Statistical analyses were performed using IBM SPSS Statistics version 25 (IBM Corp., Armonk, NY, USA). Microsoft Office Excel 2013 was used for data entry and preliminary data organization. All data were subsequently imported into SPSS and checked for accuracy prior to statistical analysis.

Non-parametric analyses were performed due to the non-normal distribution of continuous variables. The Mann–Whitney U test was used for comparisons between two independent groups, while the Kruskal–Wallis test was applied for comparisons among more than two groups. Categorical variables were compared using the Chi-square test Likelihood Ratio or Fisher’s exact test and exploratory ROC curve analysis, as appropriate.

For categorical variables with more than two categories showing statistically significant overall associations, post-hoc pairwise comparisons were performed using Fisher’s exact test on 2 × 2 contingency tables. Bonferroni correction was applied to account for multiple testing. Variables with only two categories did not require post-hoc testing, as their overall 2 × 2 test represented the only possible category comparison. Given the cross-sectional design and dichotomized outcome, ROC analyses were used for discrimination purposes only and not for predictive inference.

The ROC (Receiver Operating Characteristics) curve is a two-dimensional plot in which sensitivity is represented on the *Y*-axis and specificity on the *X*-axis. This curve is used to evaluate the performance of a model. The larger the area under the curve (AUC) (with a maximum value of 1), the better the model performance.

- AUC > 0.9—excellent;
- 0.9 > AUC > 0.8—very good;
- 0.8 > AUC > 0.7—good;
- 0.7 > AUC > 0.6—fair (acceptable);
- AUC < 0.6—the model is rejected.

Descriptive statistics were used to summarize the characteristics of the study population.

Continuous variables were expressed as mean ± standard deviation or median (minimum-maximum), as appropriate, while categorical variables were presented as frequencies and percentages.

The normality of continuous variables was assessed using the Shapiro–Wilk test. In addition, skewness and kurtosis values within the range of ±2 were considered indicative of an approximately normal distribution.

Comparisons between groups were performed using chi-square test for categorical variables. A *p*-value < 0.05 was considered statistically significant for all analyses.

Participants were classified into two quality-of-life groups (“very good” and “moderately reduced”) based on a data-driven dichotomization of the global WHOQOL-BREF score using the mean value as the cut-off point.

Given the cross-sectional design of the study, all analyses were intended to identify statistical associations between variables at a single point in time, without implying causal or predictive relationships.

### 2.13. Ethical Procedures

All participants provided informed consent. The study was approved by the Research Ethics Committee of the “Dr. C. I. Parhon” Clinical Hospital, Romania (protocol code 8358, approval date: 17 September 2024).

Given the observational cross-sectional design, all statistical analyses were performed to evaluate contemporaneous associations between variables, and no causal or predictive inferences were intended.

This manuscript was prepared and reported in accordance with the STROBE (Strengthening the Reporting of Observational Studies in Epidemiology) guidelines for observational studies.

## 3. Results

### 3.1. Response Rate

A total of 100 eligible patients completed the study. No missing data or dropouts were recorded.

### 3.2. Participant Characteristics

The study cohort consisted of 100 older patients diagnosed with cardiovascular disease. The mean age was 77.3 ± 6.86 years. The majority of participants were female (76%) and predominantly from rural areas (64%). Regarding marital status, 45% of participants were married, while the remainder were single, divorced, or widowed (Table 1).

Patients may present more than one cardiovascular condition; therefore, percentages do not sum to 100%.

Continuous variables are presented as mean ± standard deviation or median (range), as appropriate.

QoL questionnaire validation

The inter-item correlation matrix provided an overview of the degree of association between items. To ensure that there were no issues in item construction and no excessive similarity between items, Scale Reliability Analysis was applied for questionnaire validation.

The Cronbach’s alpha value was 0.893, indicating good internal consistency in relation to the accepted threshold (0.700) for validating the application of this quality-of-life assessment questionnaire.

Descriptive analysis of the QoL score in the study group showed an approximately symmetric (near-normal) distribution of values, with a variance of 12.75% across the range 57–100. The mode of the distribution (94) was close to the mean score (89.99 ± 11.47) (Table 2).

Participants with QoL scores above the mean value were classified as having very good quality of life (*n* = 61), whereas those with QoL scores below the mean value were classified as having moderately reduced quality of life (*n* = 39).

This cut-off was used solely for statistical comparison within the study sample.

QoL scores showed an approximately normal distribution.

The analysis of demographic and clinical characteristics showed a comparable distribution between the very good and moderate quality of life groups in terms of sex, age, residence and marital status, with no statistically significant differences observed (*p* > 0.05 for all variables) (Table 3).

Regarding cardiovascular comorbidities, hypertension, chronic coronary syndrome, atrial fibrillation, and heart failure showed no statistically significant differences between groups (Table 3).

Patients may present more than one cardiovascular condition; therefore, percentages do not sum to 100%.

Categorical variables were compared using Chi-square test Likelihood Ratio or Fisher’s exact test, as appropriate.

Quality of life was similar across sex, age groups, and place of residence (all *p* > 0.05). A substantial proportion of the patients in the study group reported good quality of life, with no significant differences between men and women (62.5% vs. 60.5%, *p* = 0.530), between patients aged >77 years and those aged ≤77 years (58.6% vs. 64.3%, *p* = 0.358), or between participants from urban and rural areas (69.4% vs. 56.3%, *p* = 0.139) (Figures S1–S3-Supplementary Materials).

Among patients with a perception of very good quality of life, normal cognitive status was predominant (77.0%), whereas mild and moderate cognitive impairment was less frequent (14.8% and 8.2%, respectively). In contrast, among patients reporting moderately reduced quality of life, normal cognitive status was observed in 48.7%, while moderate and mild cognitive impairments were more prevalent (33.4% and 17.9%, respectively), with a statistically significant association between cognitive status and quality of life (*p* = 0.007) (Table 4).

Emotional status was significantly associated with perceived quality of life (*p* = 0.040). In the very good QoL group, most patients had a normal emotional profile (55.7%), while 32.8% reported some depressive symptoms and 11.5% presented severe depressive symptoms. In contrast, in the moderately reduced QoL group, normal emotional status was less frequent (33.3%), whereas mild and severe depressive symptoms were more common (38.5% and 28.2%, respectively) (Table 4).

Nutritional status was also significantly associated with quality of life (*p* = 0.048). Normal nutritional status was observed in 55.7% of patients with very good QoL compared to 33.3% in those with moderately reduced QoL. The risk of malnutrition was more frequent in the moderately reduced QoL group (59.0% vs. 42.6%), while overt malnutrition was rare in both groups (1.6% vs. 7.7%) (Table 4).

Frailty assessed by the Fried phenotype was significantly associated with quality of life (*p* = 0.041), with a higher prevalence of frailty in the moderately reduced QoL group (79.5% vs. 60.7%) and a lower proportion of pre-frail individuals compared to the very good QoL group (17.9% vs. 37.7%). Similarly, frailty assessed using the Tilburg Frailty Indicator showed a strong association with quality of life (*p* = 0.001), with nearly universal frailty in the moderately reduced QoL group (97.4% vs. 70.5%). This reflects the substantial frailty burden of the hospitalized geriatric cardiovascular population (Table 4).

Functional independence was significantly associated with better perceived quality of life. All patients in the very good QoL group were independent in ADL (100.0%) compared to 92.3% in the moderately reduced QoL group (*p* = 0.029). For IADL, independence was more frequent in the very good QoL group (96.7% vs. 82.1%), with a statistically significant association (*p* = 0.017) (Table 4).

Fall risk was significantly associated with quality of life (*p* = 0.029), with a higher prevalence of high fall risk in the moderately reduced QoL group (89.7% vs. 72.1%). Finally, psychological resilience showed a strong association with perceived quality of life (*p* = 0.001), with high resilience being markedly more frequent among patients reporting very good QoL (90.2% vs. 48.7%) (Table 4).

Comparisons between groups were performed using Chi-square test Likelihood Ratio or Fisher’s exact test, as appropriate. 

Due to expected cell counts below 5 in the Fried phenotype analysis, Fisher’s exact test was applied where necessary (indicated as †).

Post-hoc pairwise comparisons were performed for variables with more than two categories that showed significant overall associations with quality of life (Table 4). Pairwise comparisons were conducted using Fisher’s exact test with Bonferroni correction for multiple testing. Among MMSE categories, participants with normal cognitive status differed significantly from those with moderate cognitive impairment (Bonferroni-adjusted *p* = 0.005). No significant differences were observed between participants with normal cognitive status and those with mild cognitive impairment (adjusted *p* = 1.000) or between participants with mild and moderate cognitive impairment (adjusted *p* = 0.487).

For GDS, MNA and the Fried frailty phenotype, no individual pairwise comparison reached statistical significance after Bonferroni correction (all Bonferroni-adjusted *p*-values ranged from 0.061 to 1.000).

**Figure 1:**
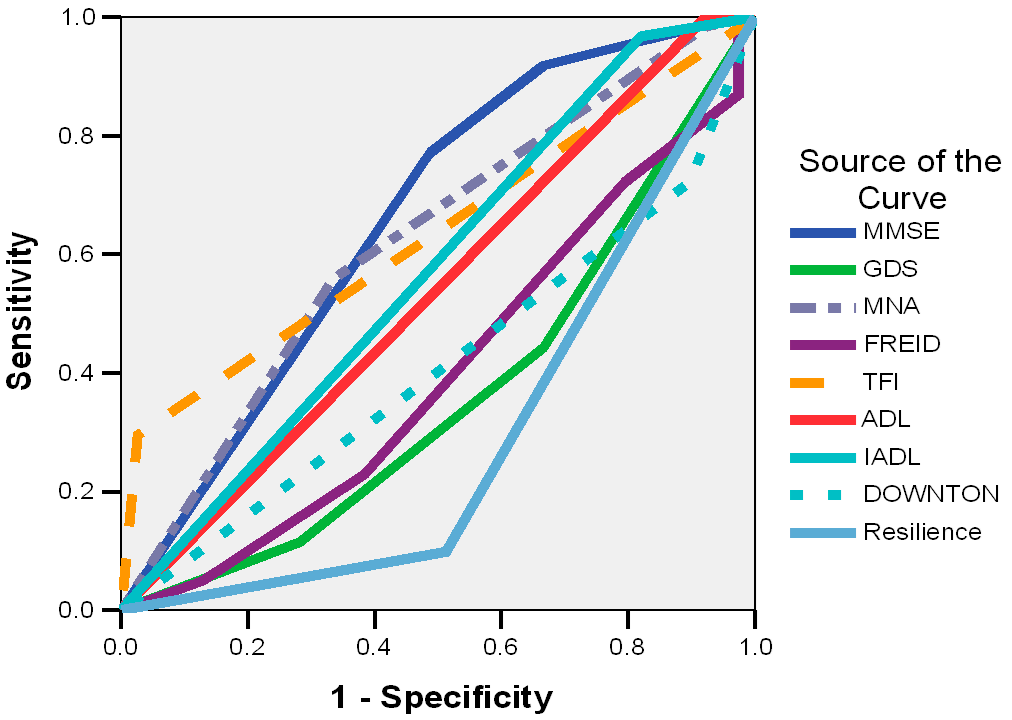
ROC analysis of variables associated with very good perceived quality of life.

ROC curve analysis was performed in an exploratory manner to evaluate the ability of the studied variables to discriminate between patients with very good and moderately reduced perceived quality of life in a cross-sectional setting. The analysis identified modest discriminative performance for MMSE (AUC = 0.660; 95% CI: 0.546–0.774; *p* = 0.007), MNA (AUC = 0.624; 95% CI: 0.511–0.736; *p* = 0.038), and Tilburg Frailty Indicator (AUC = 0.635; 95% CI: 0.527–0.742; *p* = 0.024). Other variables showed poor or non-significant discriminatory ability (AUC values close to or below 0.5), including resilience (AUC = 0.293; *p* = 0.001), indicating inverse distribution relative to the defined outcome category (Figure 1).

Area Under the Curve
Test Result Variable(s)AreaStd. Error(a)Asymptotic Sig.(b)Asymptotic 95% Confidence IntervalLower BoundUpper BoundMMSE0.6600.058**0.007**0.5460.774GDS0.3640.0580.0220.2510.477MNA0.6240.058**0.038**0.5110.736FREID0.4020.0580.1000.2880.517TFI0.6350.055**0.024**0.5270.742ADL0.5380.0600.5180.4200.656IADL0.5730.0600.2180.4550.692DOWNTON0.4120.0570.1390.3000.524Resilience0.2930.0560.0010.1820.403

The test result variable(s): MMSE, GDS, MNA, FREID, TFI, ADL, IADL, DOWNTON, Resilience has at least one tie between the positive actual state group and the negative actual state group. Statistics may be biased.

a Under the nonparametric assumption;

b Null hypothesis: true area = 0.5.

Given the cross-sectional design of the study, ROC analyses were performed to evaluate the discriminatory performance of the studied variables in distinguishing quality-of-life categories and should not be interpreted as evidence of predictive performance over time.

## 4. Discussions

The present study explored the relationships between multidimensional geriatric assessment domains and perceived quality of life in hospitalized older adults with cardiovascular disease. Our results suggest that, in this cohort of older patients with cardiovascular disease, perceived quality of life was more strongly associated with geriatric syndromes—particularly physical and psychosocial frailty—as well as with the level of resilience, whereas no measures of cardiovascular disease severity were available in this study to allow comparative assessment. This finding is consistent with recent evidence emphasizing that frailty and functional status are major determinants of cardiovascular prognosis in older adults, sometimes surpassing the predictive value of classical cardiological parameters [13].

### 4.1. Demographic Aspects

In our study, no significant associations were observed between perceived quality of life and demographic characteristics, including sex, age, marital status, or place of residence. These findings suggest that, within this hospitalized cohort of older cardiovascular patients, quality of life may be influenced more by multidimensional geriatric factors than by demographic characteristics alone. Although social determinants of health remain important contributors to health outcomes in older adults, their influence on perceived quality of life was not demonstrated in the present study, possibly reflecting the relatively homogeneous clinical profile of the study population and the limited sample size [20,21].

### 4.2. Interactions Between Cardiovascular Pathology, Frailty, and Quality of Life

In the present study, none of the cardiovascular comorbidities evaluated, including hypertension, chronic coronary syndrome, atrial fibrillation, and heart failure, showed a statistically significant association with perceived quality of life. This finding suggests that, within this cohort, subjective quality of life was more closely related to multidimensional geriatric conditions than to the presence of specific cardiovascular diagnoses [22,23,24].

In contrast, chronic coronary syndrome, atrial fibrillation, and heart failure did not show significant differences between groups. This finding may reflect the heterogeneous nature of these conditions in the older population, where clinical severity does not correlate linearly with subjective perception of quality of life. In particular, in chronic coronary syndrome, the recent literature highlights that functional impact is strongly mediated by frailty, depression, and polypharmacy, in addition to cardiovascular disease-related factors. [25,26].

Furthermore, mechanisms such as chronic inflammation, sarcopenia, oxidative stress, and impaired nutritional status are implicated both in the progression of frailty and in the evolution of chronic coronary artery disease [8,27,28,29].

It should be acknowledged that the present study did not include objective measures of cardiovascular disease severity (e.g., NYHA class, ejection fraction, symptom burden, or disease-specific severity scores). Therefore, comparisons between cardiovascular disease severity and geriatric or psychosocial factors cannot be established.

### 4.3. Cognitive Status, Frailty, and Quality of Life in Senior Cardiovascular Patients

The present findings suggest that cognitive status was significantly associated with perceived quality of life, with normal cognitive function being more frequently observed among participants reporting very good quality of life. These observations support the hypothesis that preserved cognitive function contributes to a more favorable perception of health and well-being in older adults with cardiovascular disease.

Cognitive status represents a central component of multidimensional frailty in older cardiovascular patients through the complex interaction between cognitive decline, physical vulnerability, and functional impairment.

Similarly, Hiriscau et al., in a one-year follow-up study of patients with cardiovascular disease, reported that frailty and impaired functional status were closely associated with poorer quality of life, while cognitive impairment further contributed to reduced self-care capacity and overall well-being. These findings are consistent with the associations observed in the present study [26].

In our finding, the observed association is consistent with the concept that preserved cognitive function facilitates self-care, treatment adherence, communication with healthcare professionals, and maintenance of functional independence, all of which may positively influence perceived quality of life. However, the literature shows that cognitive decline progressively contributes to frailty and reduced quality of life through impaired therapeutic adherence and self-care capacity [17,26,30].

A recent scientific statement by Ijaz et al. highlighted that cognitive frailty is increasingly recognized in older adults with cardiovascular disease as a major determinant of functional decline, poor treatment adherence, hospitalization, and adverse clinical outcomes, emphasizing the importance of routine cognitive assessment in cardiovascular care [30].

On the other hand, recent evidence describes the concept of cognitive frailty as a potentially reversible condition characterized by the coexistence of physical frailty and mild cognitive impairment, in the absence of dementia, with an important role in the progression toward disability, hospitalization, and adverse cardiovascular events [31,32,33].

Recent data suggest that cognitive frailty may be a stronger predictor of adverse outcomes than physical frailty alone, highlighting the importance of comprehensive geriatric assessment and early identification of potentially reversible factors such as depression, metabolic disorders, polypharmacy, and nutritional deficits [34,35,36,37].

### 4.4. Psycho-Emotional Status, Frailty, and Quality of Life in Senior Cardiovascular Patients

Depressive status was significantly more frequent in the group with a moderately reduced perceived quality of life, confirming the central role of psycho-emotional factors in shaping overall health perception in older cardiovascular patients. Depression is recognized as an independent predictor of reduced quality of life, being commonly associated with frailty, functional decline, recurrent hospitalizations, and increased mortality in older cardiovascular patients. The following considerations from the literature provide possible explanations for the association observed in our cohort [38,39,40].

Our findings are consistent with those reported by Deng et al., who demonstrated a bidirectional relationship between frailty and depression, suggesting that each condition may contribute to the development and progression of the other. This evidence supports the association observed in our cohort, where depressive symptoms were significantly more frequent among participants reporting only moderately reduced quality of life [40].

In recent years, the concept of psychological frailty has gained increasing attention, being defined as a state of mental vulnerability and reduced resilience, driven by diminished psychological and cerebral reserves associated with ageing. It encompasses emotional, cognitive, and motivational components and frequently coexists with physical and cognitive frailty, without representing a simple extension of either [38].

Likewise, the systematic review by Pothier et al. emphasized that psycho-social vulnerability, depressive symptoms, and chronic inflammation are closely interconnected dimensions of frailty, collectively contributing to reduced quality of life and adverse health outcomes in older adults [38].

The relationship between frailty and depression is considered bidirectional and supported by shared pathophysiological mechanisms such as chronic inflammation, oxidative stress, mitochondrial dysfunction, and dysregulation of the hypothalamic–pituitary–adrenal axis. In this context, depression may accelerate the onset of frailty by affecting nutrition, sleep, physical activity, and cognitive health, while frailty promotes social isolation, loss of autonomy, and emotional stress, thereby increasing susceptibility to depressive symptoms [39]. Recent data suggest that psycho-emotional frailty represents a distinct dimension of multidimensional frailty, insufficiently explored in clinical practice, yet with major impact on prognosis and quality of life [38,39,40,41].

Our findings are consistent with these observations and further support the importance of systematic assessment of psycho-emotional status as part of comprehensive geriatric evaluation. Early multidisciplinary interventions addressing depressive symptoms alongside frailty may contribute to improving patients’ perceived quality of life and preserving functional autonomy.

### 4.5. Nutritional Status, Frailty, and Quality of Life in Older Cardiovascular Patients

In our cohort, risk of malnutrition was significantly associated with a moderately reduced quality of life, highlighting the central role of nutritional status in maintaining physiological reserve and functional autonomy in older cardiovascular patients. Malnutrition and sarcopenia are key components of the frailty syndrome, being linked to chronic inflammation, reduced muscle mass, decreased physical performance, and metabolic vulnerability [39].

Our findings are in agreement with those reported by He et al., who analyzed two prospective cohorts and demonstrated that adverse metabolic profiles and obesity-related metabolic dysfunction were associated with accelerated frailty progression and poorer functional outcomes in older adults. These observations support the significant association identified in our cohort between impaired nutritional status and lower perceived quality of life [42].

The significant association observed in our cohort is consistent with previous reports indicating the existence of a vicious cycle between frailty and nutritional status, in which malnutrition accelerates frailty progression, while frailty further aggravates nutritional and functional decline. In addition, both undernutrition and obesity with impaired metabolic status may contribute to frailty through inflammatory pathways, insulin resistance, and fatty infiltration of muscle tissue, ultimately affecting muscle function and quality of life [41,42,43].

Similarly, Mishra et al. emphasized that frailty and metabolic dysregulation are closely interconnected through alterations in energy metabolism, chronic inflammation, and impaired muscle homeostasis, reinforcing the importance of nutritional interventions as an integral component of frailty management [43].

Taken together, these findings reinforce current recommendations regarding systematic nutritional assessment and early multidisciplinary interventions, including optimization of protein intake and physical exercise, in the management of frail older patients with cardiovascular disease.

### 4.6. Multidimensional Frailty Assessed Using Fried and Tilburg

Frailty is significantly more frequent among participants with moderately reduced perceived quality of life when assessed using both the Fried phenotype and the Tilburg Frailty Indicator. These results are consistent with previous studies reporting a strong association between frailty and quality of life in older cardiovascular patients. To better interpret these findings, the previous literature has proposed several potential explanations. According to the recent literature, frailty is a multidimensional syndrome characterized by reduced physiological reserves and increased vulnerability to stressors, being strongly associated with hospitalization, disability, and increased mortality [35,44,45,46].

Although frailty was significantly associated with poorer perceived quality of life, a considerable proportion of frail participants still reported very good quality of life. This observation highlights the multidimensional nature of quality of life in older adults, which is influenced not only by physical health but also by psychological, functional, and social resources [10,11,12,47].

An important methodological consideration is the difference between the two frailty instruments used. The Fried phenotype assesses exclusively the physical component of frailty, including reduced muscle strength, exhaustion, slow gait speed, and decreased physical activity. In contrast, the Tilburg Frailty Indicator adopts a multidimensional approach, integrating physical, psychological, and social domains [10,11,12,47,48]. The participants included in this study were hospitalized in a geriatric ward primarily due to physical health impairment, which may explain the predominance of the physical component of frailty, while psychological and social dimensions remained relatively stable. Therefore, the higher scores observed for Fried compared with Tilburg are not unexpected, given the characteristics of the studied cohort.

Our findings are consistent with previous reports; however, given the cross-sectional design, the observed associations should not be interpreted as evidence of causal relationships between frailty and perceived quality of life.

### 4.7. Functional Status (ADL, IADL), Frailty, and Quality of Life in Senior Cardiovascular Patients

In our cohort, functional independence was significantly more frequent in the group with very good quality of life, suggesting a positive association between preserved functional status and higher perceived quality of life. This direction of association was consistent across both ADL and IADL measures.

Frailty and disability are distinct but closely interrelated entities: frailty, through reduced physiological reserves and diminished resistance to stressors, progressively promotes functional decline, while disability and multimorbidity may in turn accelerate the worsening of frailty, creating a vicious cycle of biological and functional deterioration [5,49].

In this context, functional assessment remains a cornerstone of comprehensive geriatric evaluation, with prognostic value that is often superior to that of isolated cardiovascular diagnosis.

These findings further underscore the importance of preserving functional independence as an essential component of healthy ageing and quality of life in older adults with cardiovascular disease.

### 4.8. Fall Risk, Frailty, and Quality of Life in Older Cardiovascular Patients

Although fall risk was highly prevalent in the study population, it was also significantly associated with perceived quality of life, with a higher proportion of patients with moderately reduced quality of life presenting an increased risk of falls. Nevertheless, the observed trend remains clinically relevant, given that frailty, sarcopenia, and functional decline are recognized major contributors to postural instability, loss of mobility, and increased vulnerability to adverse events in older adults. Recent evidence shows that fall risk is frequently associated with chronic inflammation, reduced muscle strength, impaired balance, and cognitive decline, thereby indirectly contributing to reduced autonomy and quality of life, confirming our results [50,51].

The observed association is consistent with previous evidence suggesting that fall risk represents an important marker of global vulnerability in older adults. Because falls are closely linked to frailty, impaired mobility, cognitive decline, and loss of independence, routine assessment of fall risk should be considered an integral component of comprehensive geriatric evaluation in cardiovascular patients [50,51].

### 4.9. Psychological Resilience in Senior Cardiovascular Patients

A key finding of this study was the association between higher resilience and very good quality of life. Higher resilience was associated with very good perceived quality of life, suggesting that psychological adaptation may be related to differences in how frailty is experienced and perceived by older adults. The recent literature supports the hypothesis that resilience may represent a potentially modifiable factor associated with better therapeutic adherence and improved emotional adjustment to cardiovascular disease [52,53].

At the same time, the results indicated a high level of resilience among participants, with most resilient individuals also reporting a very good quality of life. This finding suggests that psychological resilience may be associated with a more favorable perception of quality of life. In other words, even in the presence of physical frailty, an individual’s capacity to adapt, mobilize personal resources, and maintain emotional balance may significantly contribute to a more positive perception of quality of life. Thus, our findings are consistent with the hypothesis that higher resilience is associated with better perceived quality of life, even among physically frail individuals [53].

Moreover, the results may suggest that the subjective perception of quality of life may reflect not only the severity of physical impairment, but also by the way individuals adapt to it. Quality of life is a complex construct that extends beyond physical functioning and includes dimensions such as emotional well-being, social relationships, perceived support, sense of safety, and overall life satisfaction. In this context, it is possible that participants, despite being physically frail, benefited from effective coping mechanisms, adequate family or medical support, and an increased capacity to accept and adapt to age-related changes [54]. 

The observed association may also be interpreted in the context of the “well-being paradox”, whereby older adults with multimorbidity or frailty may continue to report high levels of life satisfaction through adaptation and shifting personal priorities [55].

In addition, the hospitalization context may play an important role in interpreting these findings. Admission to a geriatric ward provides access to medical care, monitoring, and multidimensional support, factors that significantly may have contributed to an increased sense of safety and perceived support. In this sense, in-hospital interventions aimed at reducing physical frailty, combined with the preservation of patients’ psychological and social resources, may contribute to maintaining or even improving quality of life [56].

In our cohort, psychological resilience emerged as a key psychosocial correlate of quality of life, suggesting its potential role in buffering the impact of multidimensional geriatric vulnerabilities on patients’ perceived well-being. Also, a better perceived quality of life does not necessarily translate into the maintenance of a high level of psychological resilience.

At the same time, frailty, depressive symptoms, cognitive status, nutritional status, and functional independence were also significantly associated with quality of life, supporting the multidimensional nature of well-being in older cardiovascular patients.

*Limitations, Recommendations, and Directions for Future Research*

The present study has several important limitations that should be considered when interpreting the results. The cross-sectional design allows only the identification of associations at a single point in time and does not permit causal inferences regarding frailty, resilience, and quality of life. Therefore, the observed associations should not be interpreted as evidence of temporal or causal relationships between resilience, frailty, and quality of life.

The single-center design in a geriatric hospital setting may limit external validity, as the study population represents a selected group of hospitalized older adults with cardiovascular disease and higher baseline vulnerability compared to community-dwelling populations.

Although the study achieved complete data collection, this was facilitated by the inpatient setting and supervised administration of questionnaires, which may not be replicable in outpatient or community-based settings.

The high proportion of frail individuals may reflect the specific characteristics of a hospitalized geriatric population rather than a community-based sample, which should be considered when interpreting the generalizability of the findings.

An additional methodological limitation is the use of a global WHOQOL-BREF score together with its dichotomization into two categories for statistical comparisons. Although this approach facilitated group-based analyses, it may not fully capture the multidimensional structure of the WHOQOL-BREF instrument, which was originally developed for domain-specific assessment of quality of life.

A major limitation of this study is the lack of detailed assessment of cardiovascular disease severity, including functional classification, imaging parameters, symptom burden, and comorbidity indices. As a result, the independent contribution of cardiovascular disease severity to quality of life could not be evaluated, and no causal or comparative conclusions regarding its relative importance can be drawn.

Future studies should integrate validated cardiovascular severity indices (e.g., functional classification, echocardiographic parameters, disease-specific symptom burden, and multimorbidity measures) alongside comprehensive geriatric and psychosocial assessments to better delineate their relative contribution to quality of life in older adults with cardiovascular disease.

The relatively small sample size may have reduced the statistical power to detect weaker associations, particularly for demographic and cardiovascular variables. Furthermore, resilience and quality of life were assessed using self-reported instruments, which may be influenced by response bias, individual perception, and the emotional state of participants during hospitalization.

Despite these limitations, the study provides clinically relevant information regarding the multidimensional determinants of perceived quality of life in hospitalized older adults with cardiovascular disease and supports the value of comprehensive geriatric assessment in this population.

Future research should focus on prospective longitudinal studies capable of clarifying the temporal and potentially causal relationships between frailty, resilience, cognitive function, nutritional status, functional independence, and quality of life in older adults with cardiovascular disease. There is a need for standardization of the frailty definition and its integration into clinical practice through digital tools and automated early detection systems, including electronic health records and artificial intelligence-based approaches. Emerging directions also include the validation of novel biomarkers and remote assessment methods, as well as the exploration of the role of the gut microbiota, chronic inflammation, and molecular pathways involved in sarcopenia and bone loss.

In parallel, the cost-effectiveness of multimodal interventions (exercise, nutrition, deprescribing) should be evaluated, along with high-quality longitudinal clinical trials including vulnerable populations and assessing relevant outcomes such as disability, quality of life, and mortality.

Ultimately, integrating personalized geriatric care, targeted pharmacological strategies, and emerging gerontechnologies into routine cardiovascular practice may contribute to improving quality of life, reducing disability, and optimizing long-term outcomes in older adults [57,58,59,60].

## 5. Conclusions

Within the framework of comprehensive geriatric assessment, preserved cognitive function, lower depressive symptom burden, better nutritional status, lower frailty, preserved functional independence, lower fall risk, and higher psychological resilience were all significantly associated with better perceived quality of life in this cohort of hospitalized older adults with cardiovascular disease. In contrast, demographic characteristics and the presence of major cardiovascular diagnoses were not significantly associated with perceived quality of life.

Given the cross-sectional design, these findings should be interpreted as associations rather than causal relationships. Furthermore, because objective measures of cardiovascular disease severity were not available, the relative contribution of disease severity compared with geriatric and psychosocial factors could not be evaluated.

Overall, the findings support the concept that quality of life in older adults with cardiovascular disease is a multidimensional construct that extends beyond cardiovascular diagnoses alone and is closely related to geriatric, functional, psychological, and nutritional domains. Also, higher psychological resilience was significantly associated with better perceived quality of life, underscoring the relevance of psychosocial factors within comprehensive geriatric assessment and their integration into individualized, multidimensional care strategies.

These results reinforce the importance of comprehensive geriatric assessment for identifying modifiable factors associated with quality of life and support the integration of multidimensional evaluations into the routine care of older cardiovascular patients.

Future prospective longitudinal and interventional studies are needed to clarify the temporal relationships among frailty, resilience, cognition, functional status, nutritional status, and quality of life and to determine whether interventions targeting these potentially modifiable factors can improve patient-centered outcomes.

## Figures and Tables

**Table 1:** Characteristics of the study population (*n* = 100).

Variable	Category	*n* (%)/Value
Sex	Female	76 (76.0)
	Male	24 (24.0)
Age (years)		77.30 ± 6.86
Residence	Urban	36 (36.0)
	Rural	64 (64.0)
Marital status	Married	45 (45.0)
	Unmarried/divorced/widowed	55 (55.0)
Cardiovascular diseases	HTN	90 (90.0)
	CCS	66 (66.0)
	AF	16 (16.0)
	HF	47 (47.0)

Abbreviations: HTN = hypertension; CCS = chronic coronary syndrome; AF = atrial fibrillation; HF = heart failure.

**Table 2:** Global quality of life (QoL) score.

Statistic	Value
*n* (valid)	100
Mean ± SD	89.99 ± 11.47
Median	94
Variance	12.75
Skewness	−1.166
Std. error of skewness	0.241
Minimum	57
Maximum	100
Percentile 25	83
Percentile 50	94
Percentile 75	100

**Table 3:** Quality of life in relation to demographic characteristics.

Variable	Category	Very Good QoL (*n* = 61)	Moderately Reduced QoL (*n* = 39)	Chi-2 Test *p*-Value
Sex	Female	46 (75.4%)	30 (76.9%)	0.530 †
	Male	15 (24.6%)	9 (23.1%)	
Age group	<77 years	27 (44.3%)	15 (38.5%)	0.358 †
	≥77 years	34 (55.7%)	24 (61.5%)	
Marital status	Married	30 (49.2%)	15 (38.5%)	0.325 *
	Single/divorced/widowed	31 (50.8%)	24 (61.5%)	
Residence	Urban	25 (41%)	11 (28.2%)	0.139 †
	Rural	36 (59.0%)	28 (71.8%)	
HTN	Yes	55 (90.2%)	35 (89.7%)	0.599 †
CCS	Yes	40 (65.8%)	26 (66.7%)	0.543 †
AF	Yes	8 (13.1%)	8 (20.5%)	0.239 †
HF	Yes	28 (45.9%)	19 (48.7%)	0.472 †

Abbreviations: HTN = hypertension; CCS = chronic coronary syndrome; AF = atrial fibrillation; HF = heart failure. * *p* by Chi-square test Likelihood Ratio. † *p* by Fisher test.

**Table 4:** Quality of life in relation to cognitive status, emotional status, nutritional status, frailty, functional dependence, fall risk and resilience.

Variable	Category	QoL Very Good (*n* = 61) *n* (%)	QoL Moderately Reduced (*n* = 39) *n* (%)	Chi-2 Test *p*-Value
Cognitive status (MMSE)	Moderate	5 (8.2)	13 (33.4)	0.007 *
	Mild	9 (14.8)	7 (17.9)	
	Normal	47 (77.0)	19 (48.7)	
Emotional status (GDS)	Very depressed	7 (11.5)	11 (28.2)	0.040 *
	Some depressive symptoms	20 (32.8)	15 (38.5)	
	Normal	34 (55.7)	13 (33.3)	
Nutritional status (MNA)	Malnutrition	1 (1.6)	3 (7.7)	0.048 *
	Risk of malnutrition	26 (42.6)	23 (59.0)	
	Normal	34 (55.7)	13 (33.3)	
Frailty (Fried phenotype)	Frail	37(60.7)	31 (79.5)	0.041 *
	Pre-frail	23 (37.7)	7 (17.9)	
	Robust	1(1.6)	1 (2.6)	
Frailty (Tilburg Frailty Indicator)	Frail	43 (70.5)	38 (97.4)	0.001 †
	Robust	18 (29.5)	1 (2.6)	
ADL	Dependent	0 (0.0)	3 (7.7)	0.029 †
	Independent	61 (100.0)	36 (92.3)	
IADL	Dependent	2 (3.3)	7 (17.9)	0.017 †
	Independent	59 (96.7)	32 (82.1)	
Fall risk (Downton Scale)	Low	17 (27.9)	4 (10.3)	0.029 †
	High	44 (72.1)	35 (89.7)	
Resilience	High	55 (90.2)	19 (48.7)	0.001 †
	Moderate	6 (9.8)	20 (51.3)	

Abbreviations: MMSE = Mini-Mental State Examination; GDS = Geriatric Depression Scale; MNA = Mini Nutritional Assessment; ADL = Activities of Daily Living; IADL = Instrumental Activities of Daily Living. * *p* by Chi-square test Likelihood Ratio. † *p* by Fisher test.

## Data Availability

The data that support the findings of this study are available from the corresponding author upon reasonable request, due to restrictions related to participant privacy and ethical considerations.

## Supplementary Materials

The following supporting information can be downloaded at: https://www.mdpi.com/article/10.3390/jcm15156083/s1, Table S1. Calculation of WHOQOL—BREF domain scores and descriptive statistics of the study population. Table S2. Pairwise comparisons of cognitive status, emotional status, nutritional status and frailty. Figure S1. Distribution of QoL by sex. Figure S2. Distribution of QoL by age. Figure S3. Distribution of QoL by residence.
