# Supplementary material for: Multidimensional Frailty and Resilience in Relation to Quality of Life in Senior Patients with Cardiovascular Disease: An Exploratory Cross-Sectional Pilot Study
Source: J Clin Med. 2026 Aug 5;15(15):6083. doi: 10.3390/jcm15156083 (PMC13467217; doi:10.3390/jcm15156083)

Table S1. Calculation of WHOQOL - BREF domain scores and descriptive statistics of the study population

Domain	Score calculation formula	Observed range	Mean	Median	Skewness test
Physical health	(6-Q3)+(6-Q4)+Q10+Q15+Q16+Q17+Q18	14-31	24.79	26	-0.479
Phycological health	Q5+Q6+Q7+Q11+Q19+(6-Q26)	7-30	21.57	23	-0.875
Social relations	Q20+Q21+Q22	3-15	11.99	13	-1.166
Environmental relations	Q8+Q9+Q12+Q13+Q14+Q23+Q24+Q25	19-40	32.75	34	-1.178
QoL	D1+D2+D3+D4	3-100	89.99	94	-1.166

Table **S2**. Pairwise comparisons of cognitive status, emotional status, nutritional status and frailty

**Variable**	**Comparison**	**p (Fisher)**	**Adjusted p-value (Bonferroni)**
**MMSE**	Moderate vs Mild	0.162	0.487
	**Moderate vs Normal**	**0.001**	**0.005**
	Mild vs Normal	0.368	1.000
**GDS**	Very depressed vs Some depressive symptoms	0.254	0.762
	Very depressed vs Normal	0.020	0.061
	Some depressive symptoms vs Normal	0.166	0.498
**MNA**	Malnutrition vs Risk of malnutrition	0.350	1.000
	Malnutrition vs Normal	0.085	0.257
	Risk of malnutrition vs Normal	0.060	0.180
**Fried phenotype**	Frail vs Pre-frail	0.044	0.133
	Frail vs Robust	1.000	1.000
	Pre-frail vs Robust	0.443	1.000

Abbreviations: MMSE = Mini-Mental State Examination; GDS = Geriatric Depression Scale; MNA = Mini Nutritional Assessment.

p-values were calculated using Fisher's exact test and adjusted for multiple comparisons using the Bonferroni correction.

Figure S1. Distribution of QoL by sex

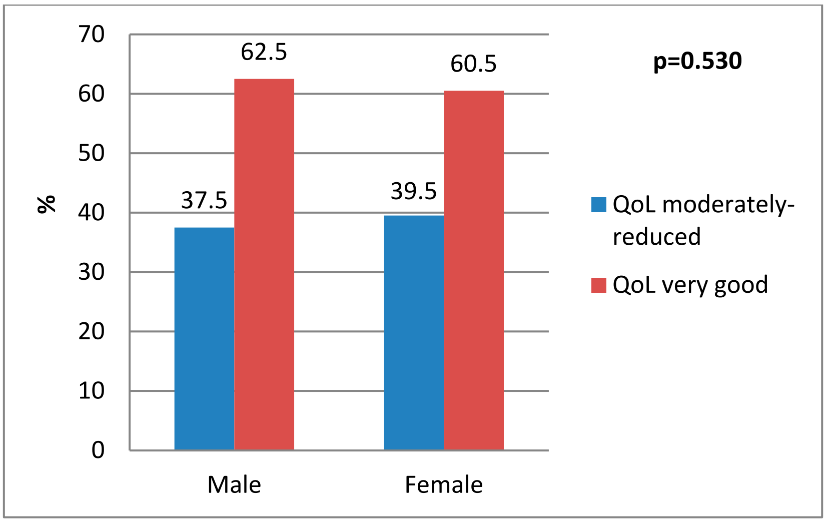

Figure S2. Distribution of QoL by age

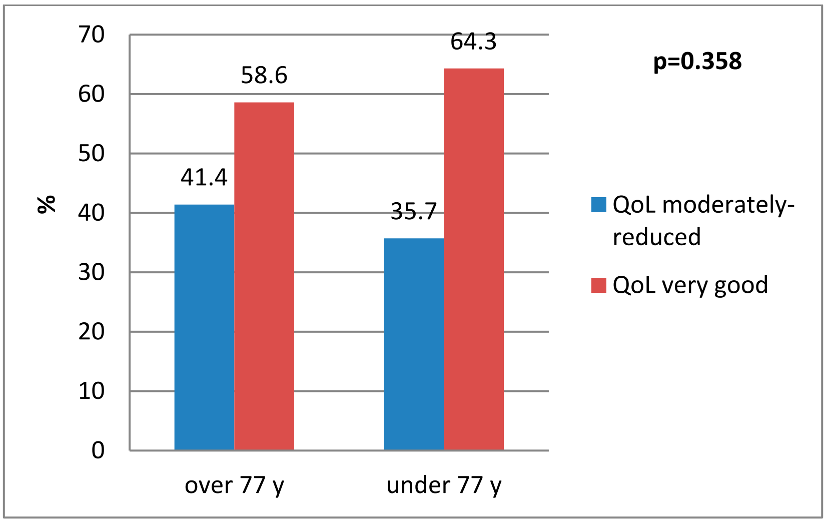

Figure S3. Distribution of QoL by residence

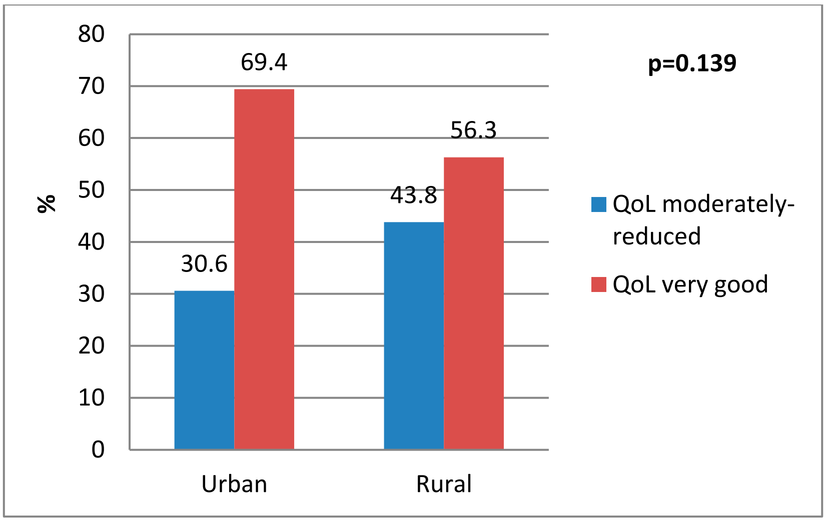

Supplement: Supplementary file 1 [file jcm-15-06083-s001.zip › jcm-4452563-supplementary.pdf]
